# Priming Intelligent Behavior: An Elusive Phenomenon

**DOI:** 10.1371/journal.pone.0056515

**Published:** 2013-04-24

**Authors:** David R. Shanks, Ben R. Newell, Eun Hee Lee, Divya Balakrishnan, Lisa Ekelund, Zarus Cenac, Fragkiski Kavvadia, Christopher Moore

**Affiliations:** 1 Division of Psychology and Language Sciences, University College London, London, United Kingdom; 2 School of Psychology, University of New South Wales, Sydney, Australia; Brain and Spine Institute (ICM), France

## Abstract

Can behavior be unconsciously primed via the activation of attitudes, stereotypes, or other concepts? A number of studies have suggested that such priming effects can occur, and a prominent illustration is the claim that individuals' accuracy in answering general knowledge questions can be influenced by activating intelligence-related concepts such as *professor* or *soccer hooligan*. In 9 experiments with 475 participants we employed the procedures used in these studies, as well as a number of variants of those procedures, in an attempt to obtain this intelligence priming effect. None of the experiments obtained the effect, although financial incentives did boost performance. A Bayesian analysis reveals considerable evidential support for the null hypothesis. The results conform to the pattern typically obtained in word priming experiments in which priming is very narrow in its generalization and unconscious (subliminal) influences, if they occur at all, are extremely short-lived. We encourage others to explore the circumstances in which this phenomenon might be obtained.

## Introduction

In recent years a substantial body of evidence has accumulated which suggests that behaviour can be unconsciously influenced or *primed* by the activation of relevant stereotypes, attitudes, traits, goals, or other concepts. Instead of, or in addition to, the normal route from conscious intentions to behaviours, individuals can be induced (it is claimed) to act socially or unsocially, walk faster or slower, behave more or less intelligently, or perceive accurately or inaccurately as a result of subtle priming influences of which they are unaware. Bargh, Chen, and Burrows, for example, asked participants in one study to read sentences containing words related to the concept *old age* and reported that a few minutes later these individuals walked more slowly down a corridor [1]. Another study reported that participants judged a hill as steeper when they were wearing a heavy backpack [2]. Equally striking, and the focus of the present research, is Dijksterhuis and van Knippenberg's report that individuals answer more general knowledge questions correctly after being asked to think about the attributes of a professor than they do after thinking about soccer hooligans [3].

Understanding the principles of behaviour change is fundamental to psychology. Consequently, demonstrations such as these call into question the standard view that the best way to effect behaviour change is through alterations to conscious beliefs and intentions. Those involved in this research have made bold statements about its importance. Bargh and Huang [4], for instance, wrote:

“Priming” refers to the passive, subtle, and unobtrusive activation of relevant mental representations by external, environmental stimuli, such that people are not and do not become aware of the influence exerted by those stimuli. In harmony with the situationist tradition, this priming research has shown that the mere, passive perception of environmental events directly triggers higher mental processes in the absence of any involvement by conscious, intentional processes…” (p. 128)

On the other hand, from the perspective of cognitive psychology, these effects are more than a little surprising. A well-established principle in traditional priming research (which commonly involves presenting words as primes to study lexical or semantic processing) is that generalization is often extremely narrow and context-specific [5]. If the priming effects of reading a word such as OLD do not transfer across changes in font or modality, then how likely is it that they transfer to something like speed of walking? The priming effects described above are unusual in this context as they imply effects which generalize very broadly. Another reason these reports are surprising is that decades of research has found that unconscious or subliminal influences on behaviour are exceptionally difficult to demonstrate [6], [7], [8], and even when replicable positive effects are shown, they tend to be over extremely short time intervals (less than a second), far shorter than the intervals involved in the studies described above, where periods of at least a few minutes are involved.

The experiments described in this article arose from an initial desire to probe more deeply into claims that the aforementioned goal priming effects are unconscious. Our intention was to use more sensitive measures of awareness to investigate the extent to which participants are truly unconscious of the influence of the prime on their behaviour in tasks where priming a concept related to intelligence (*Professor* or *Soccer hooligan*) has been reported to affect performance on a subsequent test of general knowledge. Note that the present focus on activation of other-related stereotypes (stereotypes of groups of which one is not a member) excludes studies within the ‘stereotype threat’ literature in which activation of self-related stereotypes (such as ‘African American’ for African American participants) is found to reduce academic performance [9]. In contrast to the studies reviewed here, stereotype threat research tends to assume that the effects of activating a self-related stereotype are consciously mediated and arise through raised state anxiety [10]. Another difference is that the stereotype threat effect is negative compared to a neutral control condition whereas intelligence priming can be both positive and negative.

We found it difficult, however, to replicate the basic effect of stereotype priming on accuracy in answering general knowledge questions. Our efforts to determine the reasons for this difficulty are described here, together with a Bayesian analysis of our findings [11]. This provides a precise quantitative evaluation, given a level of prior belief in the intelligence priming effect, of the posterior odds of the null hypothesis (no priming effect) against the experimental hypothesis (intelligence can be primed).

## Experiments 1–4

Dijksterhuis and van Knippenberg's [3] task involved a between-subjects manipulation. Some participants spent a few minutes describing the attributes of a typical professor, whereas others described those of a typical soccer hooligan. Specifically, they were asked to imagine a typical professor (or soccer hooligan) and to list the behaviors, lifestyle, and appearance attributes of this typical professor (or soccer hooligan). Participants then performed what was presented as an unrelated multiple-choice general knowledge test. Dijksterhuis and van Knippenberg reported that priming the stereotypes of professor (Experiments 1 & 2) or soccer hooligans (Experiment 3) affected performance on the general knowledge task, with the former leading to improved performance and the latter to worse performance. The same effect on behaviour was shown when, instead of thinking about a typical professor or soccer hooligan, participants were asked to think directly about the traits intelligence or stupidity (Experiment 4). Furthermore, there was evidence that the priming effect increased when the prime phase was longer (9 min vs 2 min).

Dijksterhuis and van Knippenberg [3] suggested that the priming effect occurs because activation of the stereotype of intelligence (in the professor compared to the soccer hooligan condition) leads participants to use more intelligent strategies for answering the questions, or motivates them to concentrate harder, or increases confidence and hence induces a beneficial response bias. The range of possible mechanisms is discussed elsewhere [12], [13].

The first four experiments reported here attempted to replicate the basic intelligence priming effect using procedures similar (Experiments 1 and 2) or identical (Experiments 3 and 4) to those used in the original study describing the effect.

### Methods

In Dijksterhuis and van Knippenberg's [3] procedure and in a subsequent replication by the same authors [14] participants simply wrote down some of the attributes of a typical professor or soccer hooligan before taking the knowledge test. In our first experiment we introduced two modifications to this procedure. First, we used an extended priming procedure in which participants watched an 8 min video clip either showing professors discussing cosmology or a documentary on soccer hooliganism, prior to (as in Dijksterhuis & van Knippenberg's procedure), requiring them to spend 5 min listing the attributes of a typical professor or soccer hooligan. On the basis of Dijksterhuis and van Knippenberg's [3] results, extending the priming stage should enhance its effect. Secondly, instead of answering general knowledge questions, participants completed questions from Raven's Advanced Progressive Matrices and they did this both before and after seeing the video. If the effects of priming are thought to be due to changes in motivation or strategy, then examining performance in a general knowledge test seems less than ideal given the binary nature of such knowledge (known/unknown). Instead, employing a test of analytical thinking and problem solving should be better suited to detecting priming effects. The method is therefore close to that of a study [15] which reported an increase in performance on a test of analytical thinking after participants listened to an audiotape of a day in the life of a professor. Participants in that study, however, were explicitly instructed to take the point of view of the professor, so the nature of the priming effect was very different.

The numbers of participants in each condition in this and the other experiments are shown in the relevant data tables. Full details of the methods can be found in the Supporting Information. At the end of each experiment participants' awareness of the purpose of the experiment was assessed via a funnel debriefing (see Supporting Information for details).

In Experiment 2 the method was the same except that we removed the video clip. The priming procedure was therefore identical to that employed by Dijksterhuis and van Knippenberg [3]. In Experiment 3 the priming procedure was the same as that in Experiment 2 but extended to 9 min, the Raven's questions were replaced with general knowledge questions, and no questions were presented prior to the priming phase, meaning that the experiment was a close replication of the method used by Dijksterhuis and van Knippenberg ([3], Experiment 4), ([14], Experiment 2), and Hansen and Wänke ([16], Experiment 2). Experiment 4 was, similarly, a close replication of the original method but with a larger sample size than any of the published studies. The priming phase lasted 5 min.

### Results

Across the experiments reported here, some participants reported in debriefing that the priming procedure might have influenced their performance in the knowledge test (see Supporting Information for details). However these reports were often vague and inconsistent with the experimental hypothesis: for instance, some participants in the soccer hooligan prime conditions reported that listing attributes about the stereotype helped them to concentrate and perform better in the general knowledge task. None of the results in any of the experiments were altered by the exclusion of these participants, and hence we report results across all participants.

To investigate the change in test performance following priming a difference score was computed for Experiments 1 and 2, by subtracting the baseline score from the post-priming score. The mean difference scores for both Experiments 1 and 2 are shown in Table 1 (effect sizes are reported in Table 2) and suggest that there was an improvement in performance in both groups, representing a benefit of practice. However, contrary to predictions, the improvement was no greater in the professor than in the hooligan condition: in fact it was the other way round in both experiments, though the mean improvement scores did not differ either in Experiment 1, *t*(38) = −1.10, *p* = .14, or in Experiment 2, *t*(14) = −0.18, *p* = .43 (because we are testing directional predictions, all reported *p* values are 1-tailed).

**Table 1:** Experimental results.

Experiment	Prime	N	Score	SD
1	Professor	20	0.4	1.8
	Hooligan	20	1.0	1.6
2	Professor	8	0.3	2.9
	Hooligan	8	0.5	2.6
3	Professor	22	50.4	12.1
	Hooligan	22	49.0	8.0
4	Professor	50	40.5	8.7
	Hooligan	50	39.6	7.0
5	Professor-similarities	25	35.6	11.4
	Einstein-differences	24	34.8	11.1
6	Professor-similarities	16	40.0	16.7
	Einstein-differences	16	33.8	10.9
7	Professor in-group	12	45.0	16.4
	Hooligan in-group	12	42.8	19.4
	Professor out-group	12	47.2	14.3
	Hooligan out-group	12	46.7	18.6
8	Incentive	20	44.5	8.3
	No incentive	20	38.6	10.1
	Professor	20	44.4	12.6
	Hooligan	20	40.1	9.5
9	Professor independent	9	53.3	30.0
	No prime independent	10	40.0	24.9
	Hooligan independent	11	50.9	28.8
	Professor interdependent	15	40.0	22.7
	No prime interdependent	12	51.7	30.1
	Hooligan interdependent	9	46.7	24.5

Note: In Experiments 1 and 2 the score is the change in number of correct answers relative to the baseline (pre-priming) test (max = 9). All other scores are percentages correct.

**Table 2:** Bayesian t-test analyses.

Experiment	Mean difference			Bayes factor	
	Effect size *d*	*t*-value	*p*-value	Cauchy	Normal
1	−0.35	−1.10	.14	2.54	1.90
2	−0.09	−0.18	.43	2.94	2.21
3	0.14	0.45	.33	4.10	3.15
4	0.11	0.57	.28	5.58	4.36
5	−0.07	−0.25	.40	4.57	3.53
7 in-group	0.12	0.30	.38	3.35	2.54
7 out-group	−0.03	−0.08	.47	3.47	2.64
8	0.38	1.20	.12	2.30	1.72
9 independent	−0.08	−0.18	.43	3.18	2.40
9 interdependent	−0.29	−0.68	.25	2.80	2.10

Note: In Experiments 7 (out-group) and 9 (independent) the predicted effect was reversed, i.e., hooligan>professor.

For Experiment 3 there was no pre-test and hence the key data are raw scores (percentages correct) on the general knowledge test. The analysis revealed that there was no significant difference between the two conditions, *t*(42) = 0.45, *p* = .33. Experiment 4 yielded no evidence of stereotype priming either, *t*(98) = .57, *p* = .28. Note that these experiments employed sample sizes comparable to or larger than those employed by Dijksterhuis and van Knippenberg [3]: their groups varied in size from approximately 10 to 32. Experiment 4 included more participants (50 per group) than any of the published studies.

Failures to obtain significant effects (null results) are often regarded as inconclusive in psychological research. Yet from a Bayesian perspective there is little difference in the status of an experimental hypothesis versus the null hypothesis, in the sense that in both cases it is meaningful to define the probability of the data given the hypothesis. A considerable body of recent research has sought to develop methods for quantifying these probabilities and, in particular, for comparing them in a likelihood ratio [11], [17]. Specifically, a “Bayes factor” is defined as the ratio of the probability of the data given the null hypothesis versus the probability of the data given the experimental hypothesis. This likelihood ratio, when multiplied by the prior odds, yields the posterior likelihood ratio of the null versus the experimental hypothesis, given the data. When this odds ratio is very small, such as 1∶10, then one can infer that the evidence strengthens the experimental hypothesis by a factor of 10, regardless of one's prior belief about the likelihood of the experimental hypothesis being true. Conversely, when the odds ratio is very large (e.g., 10∶1) then the evidence strengthens the null hypothesis by a factor of 10, again regardless of prior beliefs. We report later a full Bayesian analysis of our findings, but as a preview we note that the results of Experiments 1–4 yield Bayes factors of between 2.54∶1 and 5.58∶1 in favour of the null (see Table 2). Thus each experiment suggests an approximate trebling of the posterior probability of the null hypothesis being true, compared to the experimental hypothesis. Our subsequent analysis also includes a description of the cumulative odds ratios based on all our experimental data.

In light of these failures to obtain intelligence priming, we conducted a thorough literature review to identify all priming studies published subsequent to the original reports of Dijksterhuis and colleagues which obtained an influence of stereotype activation on some measure of knowledge or intelligence [3], [14], [18]. One study [16] employed the same task and procedure as Dijksterhuis and van Knippenberg [3] with the exception that the primes were professors and cleaning ladies, while another used professor and soccer player primes [19]. Three further reports using modifications of the original method were also identified [20], [21], [22]. The following experiments attempt to replicate the findings of these latter reports. A further study [23] obtained a nonsignificant assimilation effect (see Discussion).

## Experiment 5

The basic intelligence priming obtained by Dijksterhuis and van Knippenberg [3] is an assimilation effect in the sense that the participant's behavior purportedly comes to resemble that of the prime (professors behave intelligently, soccer hooligans unintelligently). More recently, it has been suggested that the behavioral effects of abstract social concepts such as stereotypes are not always assimilative. Instead, behavior can be shifted in the opposite direction to the activated concept. Such behavioral contrast, like assimilation, takes place following a priming procedure but only in the presence of several moderating factors assumed to trigger social comparison.

An initial suggestion was that while priming the stereotype of professors or supermodels leads to behavioral assimilation, priming exemplars such as Albert Einstein (i.e., an intelligent exemplar) or Claudia Schiffer (i.e., a purportedly low-intelligent exemplar) leads to behavioural contrast, revealed as worse performance and improved performance, respectively, in the subsequent general knowledge test [18]. The category/exemplar contrast was subsequently found, however, not to be the key variable. LeBoeuf and Estes ([22], Experiment 1) tested and found support for the alternative hypothesis that, irrespective of whether it was a category or an exemplar, a prime could produce both behavioural assimilation and contrast depending on how relevant a comparison standard it was perceived to be. Comparison relevance was manipulated by asking participants to list either similarities (high relevance) or differences (low relevance) between themselves and the prime, the idea being that listing self-prime similarities would promote consideration of the prime as a relevant comparison standard and listing self-prime differences would lead to discounting the prime as a relevant comparison standard. Different groups of participants were asked to list how similar to (high relevance) or how different from (low relevance) either professors (category prime) or Einstein (exemplar prime) they thought they were. Immediately following the priming manipulation, they were asked to answer multiple-choice general knowledge questions. The results showed that participants in the low-relevance conditions (where they listed differences) performed better than the participants in the high-relevance conditions (where they listed similarities).

LeBoeuf and Estes' interpretation of this outcome proposed that when participants in the low-relevance condition listed differences between themselves and either professors or Einstein, they activated the general trait of intelligence associated with the primes [22]. However, because they discounted the primes as relevant comparison standards, they demonstrated assimilation. Participants in the high-relevance conditions, however, listed similarities which induced them to compare themselves with professors or Einstein, leading to worse performance, and hence, behavioural contrast. The greatest difference was between the groups that listed self-professor similarities and self-Einstein differences, the latter performing substantially better than the former. In a follow-up experiment, LeBoeuf and Estes replicated the difference between these two groups ([22], Experiment 2). In a further experiment (Experiment 3) they replicated Dijksterhuis et al.'s [18] finding that performance was better after listing attributes of a category (Professor) than of an exemplar (Einstein), while again showing that generating self-Einstein differences led to better performance than generating self-professor similarities.

### Methods

The present experiment sought to replicate the pattern observed by LeBoeuf and Estes and in particular focused on the two groups that produced the biggest difference – the self-professor similarities group and the self-Einstein differences group [22]. The experiment was designed to be as close to LeBoeuf and Estes' study as possible (for full details see Supporting Information). The cover story used by LeBoeuf and Estes was that the priming manipulation and the main general-knowledge test were studies conducted by two different departments (Marketing and Psychology respectively). Their questionnaires, therefore, were printed on the corresponding departments' letterheads to strengthen the validity of the cover story.

### Results

The dependent measure was the percentage of questions answered correctly. The results are presented in Table 1. The difference between scores in the professor-similarities and Einstein-differences groups was not significant, *t*(47) = −0.25, *p* = .40, and indeed was in the wrong direction. This experiment therefore failed to replicate LeBoeuf and Estes' [22] results where there was a significant effect of prime type on performance in the general knowledge test.

## Experiment 6

What might explain the difference in results between LeBoeuf and Estes' [22] study and the previous study? We went to considerable lengths (e.g., via the cover stories) to eliminate any sort of demand effects. An obvious possibility is that demand effects were responsible for the different results in the two studies. If the participants in LeBoeuf and Estes' study – but not in Experiment 5 – detected the true purpose of the study and inferred the experimental hypothesis, this might account for the different results [24].

In Experiment 6, therefore, participants were explicitly told the experimental hypothesis and the expected direction of the effect the priming manipulation might have on their performance in the general knowledge test. Specifically, participants in the Einstein-differences condition were told that thinking about Einstein was expected to increase their performance in the general knowledge test whereas those in the professor-similarities condition were told that comparing themselves to a professor was expected to decrease their performance. If, under these conditions, it was found that there was a significant effect of the priming manipulation on the direction of performance, it would be safe to assume that demand effects did indeed have a role to play in producing these results.

### Results

The dependent measure used was, once again, the percentage of correct answers in the general knowledge test. The effect of the priming manipulation on performance (see Table 1) was once again nonsignificantly in the wrong direction, *t*(30) = −1.25, *p* = .11, suggesting that awareness of the influence of the prime on subsequent performance is by itself not sufficient to result in the behavioural effects that have been reported in intelligence priming studies.

## Experiment 7

In a further elaboration of possible moderators of assimilation and contrast, Gordijn and Stapel provided evidence that whether social comparisons lead to automatic contrast or assimilation effects on behaviour depends on whether the comparison target is categorized as an in-group or out-group member [21]. Gordijn and Stapel hypothesized that if an intergroup context is made salient, where social identity becomes more salient than one's personal identity, assimilation should occur when the activated comparison target is perceived as an in-group member. If, however, the target is perceived as an out-group member behavioural contrast should occur.

In accordance with this hypothesis, Gordijn and Stapel [21] demonstrated an in-group/out-group effect on the direction of priming. In their study, an intergroup context was made salient by telling participants, who were students at the University of Groningen, that their findings would be compared with the findings from students at the University of Amsterdam. Then, for the priming procedure, they were asked to form an impression and rate on several personality traits a person who was described as either highly intelligent or unintelligent. Also, to manipulate the social identity of the comparison target, the target person was described as either a former student from the University of Groningen (i.e., in-group) or from the University of Amsterdam (i.e., out-group). Subsequently, participants were given a purportedly unrelated general knowledge test. In line with Gordijn and Stapel's prediction, priming with in-group comparison targets led to behavioural assimilation (i.e., enhanced performance with the intelligent target and poorer performance with the unintelligent target) while out-group comparison targets led to behavioural contrast (i.e., poorer performance with the intelligent target and enhanced performance with the unintelligent target). This implies that the social identity (in-group vs. out-group) of a comparison target can moderate the direction of automatic behaviour.

In a further effort to obtain an intelligence priming effect, we attempted to replicate Gordijn and Stapel's study. Their article [21] was retracted after the completion of Experiment 7 and hence their data can be given no evidential weight. Nevertheless, the hypothesis they put forward is a reasonable one and thus we report Experiment 7 in relation to that hypothesis. Note that another report on intelligence priming has also recently been retracted [25]. An intergroup context was made salient by informing participants (students from University College London [UCL]) that their results would be compared with the results of students from Birmingham University, where the same experiment was being run. The priming manipulation involved asking participants to form an impression of a person who was described as either a professor or soccer hooligan and who was either a former student from UCL or from Birmingham University. Thus assimilation to the comparison target is expected when the target is categorized as an in-group member, whereas contrast should occur when the target is perceived as an out-group member.

### Methods

The experiment had a 2×2 design with intelligence of the target (professor vs. hooligan) crossed with social identity (i.e., in-group vs. out-group). The procedure closely followed that of Gordijn and Stapel's Experiment 2 [21]. A slight modification was made to the intelligence manipulation: the comparison target was either described as a professor or a soccer hooligan instead of being described as a highly intelligent or unintelligent person. In addition, a post-experiment questionnaire was employed to assess awareness of the link between the priming manipulation and the general knowledge test. For full details see the Supporting Information.

### Results

We conducted a 2×2 ANOVA on the percentage of correct answers with target intelligence (professor vs. hooligan) and social identity (in-group vs. out-group) as the between-subjects factors to determine whether the priming manipulation affected performance in the general knowledge test. There was no main effect of intelligence, *F*(1, 44) = 0.08, *p* = 0.78, or identity, *F*(1, 44) = 0.38, *p* = 0.54, nor, crucially, an interaction, *F*(1, 44) = 0.03, *p* = 0.87. Means are reported in Table 1. Simple effects analysis revealed no significant effect in either the in-group condition, *t*(22) = 0.30, *p* = 0.38, where forming an impression of the professor comparison target was expected to boost performance in the general knowledge test compared to the soccer hooligan target, or the out-group condition, *t*(22) = −0.08, *p* = 0.47, where forming an impression of the professor target was expected to impair performance. Once again, we found no effect of the priming manipulation on general knowledge test performance.

## Experiment 8

In light of these failures to detect any reliable priming effects, Experiment 8 aimed to explore whether it is possible to affect general knowledge test performance by a different manipulation, monetary incentives. It is well known that incentives and rewards can motivate subjects to participate in experiments in a more effortful and considered fashion and to improve their performance against some standard [26]. In a meta-analysis of published studies, incentives were found to have a medium-sized effect on IQ performance [27] and effects of incentives on a range of measures of educational performance in classroom settings have been reported [28]. Thus, incentives may motivate participants to employ more cognitive effort in a general knowledge task. The present experiment therefore tests the hypothesis that if intelligence priming can improve general knowledge ability through increased motivation, then so too should explicit reward.

The experiment included 4 groups. Two of these performed the general knowledge test under incentives or no incentives with no priming phase. Two further groups performed the test after the standard positive (*professor*) or negative (*hooligan*) priming induction in a further attempt to replicate the basic intelligence priming effect.

### Methods

Participants were given a general knowledge test similar to those of previous experiments. For participants in the incentive condition, an initial endowment of 50 pence was allocated and an extra 20 pence was earned for every question answered correctly. Participants in the no-incentive condition were asked to solve the general knowledge test with no monetary reward except for the initial 50 pence payment. In the incentive and no incentive groups we also recorded the time participants spent on each question.

### Results

Performance in the general knowledge test differed significantly between the incentive and no-incentive conditions, *t*(38) = 2.00, *p* = 0.026, *d* = 0.63, but not between the professor and hooligan conditions, *t*(38) = 1.20, *p* = 0.12, *d* = 0.38 (see Table 1), although the priming effect was in the right direction. Participants spent marginally longer answering each question in the incentive than in the no incentive condition: the mean of the median response times was 9.01 sec in the no incentive condition and 10.7 sec in the incentive condition, *t*(38) = 1.59, *p* = 0.06, *d* = 0.50. Thus an overt motivator such as monetary incentive can reliably influence performance on the sort of general knowledge test employed in the standard intelligence priming procedure, and the results confirm that with our general methods and participant population it is possible to observe reliable changes in general knowledge performance. However, once again no effect of the professor/hooligan prime was obtained.

## Experiment 9

Yet another variation on the basic intelligence priming effect was reported by Bry, Follenfant, and Meyer [20]. These researchers considered the moderating influence of self-construal on contrast and assimilation. ‘Self-construal’ refers to the way in which the self is mentally represented and can vary from independence, when the individual thinks of herself as unique, autonomous, and distinct from others, to interdependent, when she conceptualizes herself as connected to others and part of a larger group. Bry et al. proposed that assimilation to an intelligent or unintelligent prime would occur under interdependence and that contrast would occur under independence, and obtained experimental support for this pattern.

To test this hypothesis, participants in Bry et al.'s Study 2 [20] initially rated themselves in relation to a series of statements designed to evoke independence (e.g., “I am unique – different from others in many respects”) or interdependence (e.g., “to understand who I am, you must see me with members of my group”). This was followed by a priming phase in which participants answered questions about a series of faces. Bry et al. activated the ‘dumb blonde’ stereotype by including a large proportion of faces of blonde-haired females. Finally, participants answered general knowledge questions. Bry et al. found reliable assimilation (worse performance after priming the stereotype Blonde than after no priming) under the interdependent self-construal and contrast (better performance after priming the Blonde stereotype than after no priming) under independence.

We closely replicated Bry et al.'s procedure [20], but used the professor/soccer hooligan stereotypes as well as a no-prime control condition. Specifically, participants in the primed groups answered questions about pictures of professors or soccer hooligans. For full details see the Supporting Information.

### Results

Bry et al. [20] only obtained a priming effect on a subset of moderately difficult questions in their general knowledge task. The percentage of correct answers in their study for these questions was approximately 40%. Hence, we selected test questions which on average showed a similar level of correct answers. In our preliminary analysis, we estimated the mean of correct responses of each of the twenty questions and ranked them into 4 groups of 5 questions according to their difficulty. Our analysis revealed that the most difficult questions on our questionnaire were answered correctly at a level similar (47% correct) to those in Bry et al.'s [20] analysis.

A 2 (self-construal: independence vs. interdependence)×3 (professor prime, hooligan prime, no prime) ANOVA was performed on data from these difficult questions (see Table 1). The analysis revealed no main effect of self-construal, *F*(1, 60) = 0.09, *p* = . 77, or of prime, *F*(2, 60) = 0.07, *p* = .94. The interaction was also not significant, *F*(2, 60) = 1.23, *p* = .30. Indeed the means for the professor and hooligan prime conditions were in the opposite direction to those observed by Bry and colleagues. Simple effects analysis revealed no significant effect in either the interdependent self-construal condition, *t*(22) = −0.68, *p* = .25, where forming an impression of the professor comparison target was expected to boost performance in the general knowledge test compared to the soccer hooligan target, or the independent condition, *t*(18) = −0.18, *p* = .43, where forming an impression of the professor target was expected to impair performance. Note that the standard deviations reported in Table 1 are much larger (though in line with those reported by Bry et al.) than in previous experiments as a result of the small number of questions analysed. The same pattern of nonsignificant results was obtained, however, when we analysed all 20 questions in the test.

## Bayesian Comparison of the Null and Experimental Hypotheses

As noted earlier, traditional statistical methodology regards failures to reject the null hypothesis as inconclusive. Indeed, in Fisherian statistics “every experiment may be said to exist only in order to give the facts a chance of disproving the null hypothesis” ([29], p. 19). The null hypothesis significance testing approach has been severely criticized, however, because it invites a number of fallacious inferences, such as that rejection of the null hypothesis is equivalent to affirmation of the experimental hypothesis, that *p*-values provide measures of evidential support, and that they represent the probability that the null hypothesis is true [30]. In response to these concerns, Bayesian approaches have been developed which regard the experimental and null hypotheses as on an identical footing in terms of their ability to be evidentially supported or disconfirmed and which, of particular importance, allow one to evaluate evidence *for* the null hypothesis from a given experiment or set of experiments [11], [17]. See [31] for a recent application of these methods in another domain where unconscious influences have been hypothesized.

We used the method described in [11] to compute a Bayes factor for each experiment, defined as the ratio of the probability of the data given the null hypothesis to the probability of the data given the experimental hypothesis. To compute these factors, it is necessary to specify the form of the distribution of possible effect sizes under the experimental hypothesis. Two candidates are the normal and Cauchy distributions. The Cauchy prior makes weaker assumptions about the likely effect sizes under the experimental hypothesis and is therefore preferred, although we report results based on both. Although the experiments were conducted for the purpose of testing directional hypotheses, we report two-tailed Bayes factors because – as the reports of assimilation and contrast indicate – an effect in either direction could be interpreted as evidence of priming.

The Bayes factor for a given experiment, when multiplied by the prior odds, yields the posterior likelihood ratio of the null versus the experimental hypothesis, given the data. It is important to note that the interpretation of a given Bayes factor is independent of one's predispositions regarding the *ex ante* probability of the experimental hypothesis being true. Suppose a researcher is strongly predisposed to believe that intelligence can be primed in the situations studied here; perhaps her belief is that the experimental hypothesis is 10 times more likely than the null hypothesis (odds null∶experimental of 1∶10, equivalent to a probability of *p* = .91 of the experimental hypothesis being true). After observing data from an experiment which yields a Bayes factor of 2∶1, this researcher should rationally adjust her odds to be 2∶10 ( = 1∶5). Consider in contrast another researcher who is strongly predisposed to doubt that intelligence can be primed; let us imagine that he believes that the experimental hypothesis is 10 times less likely than the null hypothesis (odds null∶experimental of 10∶1, equivalent to a probability of *p* = .09 of the experimental hypothesis being true). After observing the same data from an experiment with a Bayes factor of 2∶1, this researcher should adjust his odds to be 20∶1. In both cases the posterior odds have changed by the same factor of 2∶1.

Table 2 presents the effect sizes, *t* and *p*-values, and Bayes factors for each relevant comparison. We treat the in-group and out-group contrasts in Experiment 7 as if they were independent experiments, and similarly for the independent and interdependent contrasts in Experiment 9 (where in addition we ignore the no prime groups). The table shows that for each experiment, the null gains at least twice as much support as the experimental hypothesis, and for 6 of the contrasts (Cauchy) the Bayes factor is greater than 3. A Bayes factor of between 1∶1 and 3∶1 is taken to provide ‘anecdotal’ evidence in favour of the null, whereas factors of between 3∶1 and 10∶1 provide ‘substantial’ support [32]. A meta-analysis across all experiments (with the data *z*-transformed within each experiment) yields a Bayes factor of 12.1, representing strong support for the null.

## Discussion

We investigated here the claim that priming the concept of intelligence can influence behaviour. This claim is important not only because of its theoretical implications [4] but also because of the possibility that performance in significant applied settings, such as taking classroom exams, can be boosted by unconscious priming [33]. However the results reported here suggest that priming the concept ‘Professor’ (versus ‘Soccer hooligan’) confers no advantage in answering general knowledge questions. Activating the concept or goal of intelligence via priming a related concept does not appear to increase intelligence in these experimental conditions. The Bayesian analysis reported here suggests that considerable confidence can be placed in this null hypothesis.

Why might we have obtained results so different from previous published findings? One possibility is that our priming manipulations were simply ineffective. This however seems unlikely. The stereotypes (professor/soccer hooligan) are well entrenched in the minds of our participant population, and we closely followed the original protocols in the published studies. It is noteworthy that the types of priming manipulation reported previously to be successful have varied quite widely in their format; for example, while Dijksterhuis and van Knippenberg's participants [3] considered the attributes of the prime target for several minutes, LeBoeuf and Estes [22] observed a priming effect with the standard attribute-listing methodology when participants merely listed 3 attributes, which may have taken only a few seconds. Participants in Bry et al.'s study [20] simply reported the hair color of a series of individuals shown in pictures. Thus studies reporting positive priming effects have done so across wide variation in the priming procedure.

A second possibility is that the knowledge tests we used were different in some subtle but key way from those employed elsewhere. Against this, however, is the fact that we modelled our tests on those used previously (in Experiment 5, for instance, we used many of the same questions that LeBoeuf and Estes had employed) and we ensured similar levels of overall difficulty. In addition we used a wide range of test questions, including non-overlapping tests across our experiments, as well as different forms of assessment (Raven's matrices). Moreover we were able to affect performance via monetary incentives (Experiment 8). Thus the general knowledge test was not entirely insensitive.

A third possibility is that previous studies have yielded priming effects because compliant participants were able to infer, and hence behave in accordance with, the experimenter's hypothesis [24]. Although such a possibility would undermine the theoretical significance of the effect, it would nonetheless imply that it should be replicable. Perhaps for some reason our participants did not respond in this way to the task demands. Our early failures to obtain intelligence priming encouraged us to take this explanation seriously, especially in light of other recent evidence: in another priming situation, it was reported that participants judged a hill as steeper when they were wearing a heavy backpack [2], but a later study found evidence that this is an artefact of compliance by participants to the perceived experimental hypothesis [34]. The results of Experiment 6, however, provide little support for this possibility within the intelligence priming task. An explicit instruction as to the nature of the experimental hypothesis failed to yield priming. Nevertheless, subtle differences between the present experiments and previous ones may have affected participants' detection of and compliance with task demands. The method employed in Experiment 6 to induce compliance may have been inadequate, and the failure to induce priming in that experiment does not categorically rule out the possibility that participants in previous demonstrations of intelligence priming were reacting to the perceived task demands. Future research could profitably explore alternative methods to vary compliance levels.

A fourth possibility is that our experiments lacked sufficient power to detect small but real intelligence priming effects. The Bayesian analysis reported above takes sample sizes into account and hence its conclusion indirectly reflects experimental power: for a given *t*-statistic, the Bayes factor increases with sample size. Conventional power analysis therefore becomes redundant, but we comment briefly on power nonetheless. Published effects have been uniformly large to very large in terms of effect size. Effect sizes in the original experiments reported by Dijksterhuis and van Knippenberg [3] ranged from 0.83 to 1.35. In Dijksterhuis and van Knippenberg's [14] Experiment 2 the effect size (Cohen's *d*) was 2.11, in Hansen and Wänke's [16] Experiment 2 *d* = 0.76, and in Nussinson et al.'s [19] Study 3 *d* = 0.84. In LeBoeuf and Estes' [22] Experiment 2 the comparison between scores in the self-professor similarities and self-Einstein differences groups had *d* = 0.72. In Bry et al.'s [20] Study 2, the priming effect size for the independent self-construal was *d* = 0.88 and for the interdependent self-construal was *d* = 0.79. These are strikingly large effects (mean *d* = 1.05, median = 0.86). Naturally, some of the individual experiments or comparisons reported here had low to moderate power. However, Experiment 4 was specifically conducted to provide a near-exact replication of the published method with a large sample size (larger than used in any of the published studies), and that experiment alone had ample power to detect effects of the magnitude noted above: it had power of 1 - β = .99 to detect a large effect (i.e., *d* = 0.8) and power of .80 to detect a medium-sized effect (i.e., *d* = 0.5). Across all the relevant comparisons (Experiments 1–5 and 8, the in-group/out-group contrasts in Experiment 7, and the independent/interdependent self-construal contrasts in Experiment 9), the cumulative β (i.e., the probability of all comparisons failing to detect a true large effect, *d* = .8, one-tailed) is approximately *p* = 10^−7^. The equivalent value for failing to detect a true medium effect (*d* = .5) is *p* = .002. Hence whatever the reason for our failure to obtain priming effects, low power does not seem a plausible explanation.

### Are the published effects false positives?

The evidence marshalled above against these four hypotheses suggests that they fail to provide a compelling explanation of the difference in the outcomes of the previous studies and those reported here. A fifth and final possibility is that some or all of the published results on intelligence priming were false positives. Is this a more plausible explanation? One notable feature of the published studies is the number of experiments whose results are statistically nonsignificant at the conventional *p* = .05 level. For example, in Dijksterhuis et al.'s [18] Study 1, described previously, there were four different primes: the stereotypes professors and supermodels, and the exemplars Albert Einstein and Claudia Schiffer. Although there was a reliable difference in general knowledge test scores between groups primed with the exemplars, the difference between groups primed with professors and supermodels was not significant. Rather than interpreting this as a failure to replicate the basic intelligence priming effect, Dijksterhuis et al. [18] noted that the effect was in the expected direction and concluded that stereotype priming can indeed influence test scores. Similarly, Schubert and Häfner [23] obtained a nonsignificant difference between groups expected to show the standard assimilation effect, but again did not interpret this as casting doubt on the existence of intelligence priming. We have described in detail one of Gordijn and Stapel's [21] studies (Experiment 2), but in another one (their Experiment 1) the behavioural assimilation effect under an intergroup focus was nonsignificant. It is noteworthy that another experiment by Dijksterhuis et al. [18] (Study 2) failed to replicate Bargh et al.'s [1] finding that priming the stereotype of elderly people can affect walking speed. On the grounds that the effect was in the expected direction, Dijksterhuis et al. again interpreted their data as supporting the experimental hypothesis.

In each of these cases the hypothesis was that priming would induce a change in test scores, but the authors did not take the nonsignificant result they obtained as evidence against the hypothesis. Rather, because the effect was numerically in the expected direction in each case, they took it as supportive evidence. These examples suggest that a considerable degree of confirmation bias [35] is clouding objective interpretation of the evidence.

Another bias which may contribute to the publication of false positive effects is the inappropriate employment of what have been labelled ‘researcher degrees of freedom’, the post hoc selection of data or analysis methods [36]. If the researcher distributes many questionnaires to participants in a session, for example, but only publishes the findings from a subset of the questionnaires (the ones which yield significant effects), then the true *p* value is likely to be considerably larger than .05 and the reported results are more likely to be false positives. One way to guard against this is to distinguish between exploratory studies on the one hand which are undertaken to explore a novel hypothesis, and confirmatory ones on the other hand which are undertaken solely with the purpose of replicating the findings of exploratory studies. Ideally, the details of planned confirmatory studies should be decided beforehand (and perhaps even made public) to completely eliminate the possibility of unintentional use of researcher degrees of freedom [37].

Is there any evidence of post hoc selection of data or analysis methods in the intelligence priming studies under consideration here? Recall that Bry et al. ([20], Study 2) reported evidence of assimilation under the interdependent self-construal and contrast under the independence self-construal (although Experiment 9 failed to replicate this pattern). As noted previously, the interaction reported by Bry et al. [20] pertained to moderately difficult questions, and not to difficult ones. For difficult questions, there was no interaction in the data. Is it possible that by selecting and analysing subsets of questions at different levels of difficulty, Bry and colleagues were permitting themselves a researcher degree of freedom which might have inflated the false positive rate? It is instructive to consider a recent replication of this study which Bry and colleagues reported [38]. Bry et al. [38] again obtained this pattern, namely of an interaction between prime and construal. However, the reported interaction was significant for difficult questions and not for moderately difficult ones, exactly the opposite to what was reported by Bry et al. [20]. This pattern, at the very least, is suggestive of inflation in the false positive rate as a result of inappropriate and *ex post* selection of the data to be analyzed.

It is important to emphasize that false positives could arise entirely from unintentional practices on the part of the researcher. As an illustration [39], imagine that an experimenter runs a series of experiments but makes errors in some of his statistical analyses, something which has been shown to occur in a surprisingly high proportion of research reports in psychology [40]. If the researcher checks his analyses more carefully when an unexpected null result is obtained than when an expected positive result is obtained, then the false positive rate will be inflated. An analytic error in a false null result is more likely to be detected than one in a false positive result.

False positives can also arise, of course, because of intentional malpractice by researchers. The retraction of two papers on intelligence priming [21], [25] means that the findings reported in those studies must be treated as false positives.

A final form of biased research practice is publication bias. There has been renewed debate recently around the possibility that the research environment in experimental psychology may unintentionally lead to inflation in the reporting of false positives. Several factors have been identified as possible contributors to such inflation, most notably the file-drawer problem, whereby failed replications are less likely to be published than successful ones [39], [41], [42], a problem which appears to be getting worse [43], and the increased prevalence of short-format journal articles, which tend to include small numbers of studies or studies with small sample sizes [44]. We cannot know how many failed replications of the basic intelligence priming effect might exist in researchers' file drawers, but the PsychFileDrawer.org website includes details of two failed replications [45], [46] and notes that one of these studies was conducted in 2001, although it was not made publicly available until 2012. Thus we have evidence that for a decade, there existed at least some degree of bias in the published literature on intelligence priming.

It was noted earlier that the reported effect size for the intelligence priming effect across published studies is surprisingly large (*d*≈1). There are two points to make about this. First, it implies that what is supposed to be a subtle effect is in fact larger than many standard effects in cognitive psychology (and it also makes the present failure to replicate the effect more puzzling as a large effect should be more immune to minor procedural variations). To put this in perspective, a meta-analysis of studies of stereotype threat estimated a mean effect size of 0.26 [47]. Secondly, it is not open to a defender of intelligence priming to claim that the true effect is notably smaller and that the published studies have, merely by chance, obtained larger effects. The reason this position is untenable is that if the true effect is real but smaller (say, *d* = 0.2), then the published literature must indicate publication bias. Given the moderate power of the published experiments, the number of experiments which successfully obtained the effect should have been very small (only 1 or 2), even if intelligence priming is a true effect [39]. The fact that the majority of published studies reported an effect could in that case imply only one thing, namely that they are a biased sample and that numerous failures to obtain the effect have, for whatever reason, not been published.

Our suggestion that some or all of the published effects are false positives might appear implausible given the sizable number of published reports. Yet many of those reports have examined interactive effects on intelligence priming such as the effects of self-construal, category versus exemplar priming, and so on and did not include control conditions replicating the basic priming effect. How many independent experiments have reported a basic priming effect of attribute listing (describing the characteristics of a typical high-intelligence versus low-intelligence individual) on general knowledge performance? Dijksterhuis and van Knippenberg's [3] initial report included 4 experiments and their later report ([14], Experiment 2) added a further replication. Nussinson et al. ([19], Study 3) reported a significant difference between groups primed with professors versus soccer players and in Hansen and Wänke's ([16], Experiment 2) replication the primes were professors and cleaning ladies. In contrast, Dijksterhuis et al. ([18], Study 1) obtained a nonsignificant difference between groups primed with professors and supermodels. Thus successful intelligence priming effects using the basic procedure have been obtained in 7 experiments reported in only 4 articles from 3 research groups across a period of 14 years. A total of 6 experiments (Experiments 3, 4, and 8 from the present series, Dijksterhuis et al.'s Study 1 [18], and [45], [46]) have failed to obtain the effect with the basic procedure.

## Conclusion

We do not deny outright the possibility of unconscious influences on behavior, and obviously the present experiments only relate to one particular type of priming and one particular behavior. However the present results are consistent with many other examples where claims of unconscious influences have not withstood subsequent scrutiny: for a small sample of relevant instances, see [31], [48], [49], [50], and for a comprehensive recent review see [51]. It is never possible to recreate exactly the conditions of previous experiments but if intelligence priming effects are as important as some claim them to be (and as large), then they ought to withstand minor variations in procedure, otherwise we have little prospect of understanding their basis and it is unclear why they should be afforded substantial theoretical significance. From the perspective of the cognitive priming literature, in which such effects tend to be very narrow and context-specific, the absence of an effect in the present studies is entirely unsurprising. Of course the typical procedures employed in cognitive and social priming experiments are often very different and they tend to focus on different forms of awareness (awareness of the prime in the former case and awareness of the influence of the prime in the latter case; see [51] for an extensive discussion), but the convergence of findings is noteworthy. The current results are also consistent with the view that conscious thoughts are by far the primary driver of behavior [52] and that unconscious influences – if they exist at all – have limited and narrow effects.

The theoretical and practical implications of intelligence priming are considerable. It is important that these elusive effects are studied further in independent laboratories to try to determine under what conditions they might be obtained.

## Supporting Information

Supporting Information S1 (DOCX)
