# Supplementary material for: Priming Intelligent Behavior: An Elusive Phenomenon
Source: PLoS One. 2013 Apr 24;8(4):e56515. doi: 10.1371/journal.pone.0056515 (PMC3634790; doi:10.1371/journal.pone.0056515)
Supplement: Supporting Information S1 — (DOCX) [file pone.0056515.s001.docx]

Supporting Information

**Experiment 1**

*Methods*

Participants. A total of 40 participants (28 female and 12 male) were recruited. Half were psychology students. The age of the participants ranged from 19-79 years, with a mean of 27.0 years (*SD* = 12.8). Furthermore, 12 of the participants (6 in each condition) were tested in Sweden. These participants received the question in the priming procedure in both English and Swedish, and answered the question in Swedish.

Ethics statement. This and all other experiments reported here was approved by the UCL Division of Psychology and Language Sciences Ethics Committee. Participants gave written informed consent.

Materials: IQ test. The second half of Raven’s APM Set II was used, namely the last 18 problems (numbers 19-36). Two sub-sets consisting of 9 problems each were created. The problems in Raven’s APM become progressively harder so one test consisted of the odd-numbered questions and the other of the even-numbered ones. Each problem was presented on a separate A4 page, and the 9 problems that made up each test were stapled together into a booklet. A separate answer sheet was also provided. Which sub-set was used in the pre-test and which was used in the post-test were counterbalanced.

Priming procedure. For the priming procedure a PowerPoint presentation was created, on which all instructions for this phase as well as a video-clip, were presented. The instructions emphasized that the priming procedure was unrelated to the IQ tests, which were paper based. Depending on the priming condition, the video-clip showed either professors discussing parallel universes or a documentary on soccer hooligans. Given that the use of exemplars has been shown to elicit behavioural contrast rather than assimilation, these video-clips were chosen because they involved of several members of the stereotype groups rather than presenting individual recognisable exemplars. The video-clips were each approximately 8 min in length.

Procedure. The experiment was presented as two independent pilot studies that were being conducted to collect data for two forthcoming experiments, one assessing the difficulty level of some intelligence tests and the other involving watching a video-clip and answering some questions to provide ideas for a future study. Participants were initially given the first IQ test together with written instructions, for a period of 10 min. Upon completion of the baseline test, participants were presented with the priming procedure, introduced as the second pilot study. Participants were instructed that they would be presented with a video-clip (approximately 8 min long) and would subsequently be asked some questions relating to the theme of the film. They were also advised that this was not a memory test and that they did not need to remember any details from the film. Afterwards they were asked to imagine a typical soccer hooligan or professor (depending on condition) and were given 5 min to list lifestyle, behavior, and appearance attributes of this person.

Following this, participants completed the second IQ test. When the experiment was completed a funnel debriefing was administered to ensure that any participant who may have been suspicious as to the real purpose of the study (i.e., aware of the relationship between the two supposedly unrelated pilot studies) could be identified.

*Results*

The data collected in this study were the numbers of correct answers on each IQ test, providing a baseline score as well as a post-priming score. The mean baseline scores were similar between the two priming conditions (*M* = 2.5 for the professor prime, *M* = 2.6 for the hooligan prime).

**Experiment 2**

*Methods*

Unless otherwise noted, the method was identical to that of Experiment 1. The same IQ tests were used as in Experiment 1. For the priming procedure a PowerPoint presentation was created, including the instructions and the question for the writing task. The total time for the priming procedure was 5 min.

Participants. Sixteen participants (10 female and 6 male, age range 18-60 years, mean 32.6 years, *SD* = 13.7), 8 in each condition, were recruited using availability sampling. All participants were tested in English.

*Results*

The mean baseline scores were somewhat different between the priming conditions (*M* = 3.9 for the professor prime, *M* = 2.5 for the soccer hooligan prime).

**Experiment 3**

*Methods*

Participants. Forty-four students from University College London (UCL) participated in the experiment. There were 10 males and the mean age was 21.8 (*SD* = 3.7).

Materials. Participants were given a booklet that contained the instructions for the priming phase. A questionnaire testing participants’ judged level of concentration, effort, confidence, strategy on the general knowledge test, and level of awareness was included at the end of the study.

The title ‘Experiment 1’ was written at the top of the page of instructions for the priming task. Participants were given 9 min to list the behaviors, lifestyle, and appearance of a typical professor or soccer hooligan. The next page instructed participants to attend to the computer screen for the instructions of ‘Experiment 2’, which was the general knowledge task. The computer displayed both the instructions and the general knowledge questions. The instructions (from [[3](#_ENREF_3)]) shown on the screen were: “You will be given 42 general knowledge questions to do. Your data will be used for developing a ‘general knowledge’ scale currently being developed by the Personality Research Group. We have 5 different sets of this ‘general knowledge’ test which differ in difficulty and we would like to test the differences in difficulty between these. You are being given the *most difficult* test among the 5 sets. When you press the ‘space bar’ on the keyboard each question will appear on the screen. On the Answer Sheet given, indicate the answer for the question by choosing one out of four options. An example of how to indicate the answer is shown on the Answer Sheet. You must answer every question and if you do not know the answer for the question try your best to guess the answer. There is no time constraint.”

The general knowledge questions (a pilot study indicated that students answered about 50% correctly) were drawn from the game Trivial Pursuit, Family Edition. Half of the participants received the general knowledge questions in a reverse order.

At the end of the experiment a questionnaire was administered to test participants’ awareness of any influence of the priming procedure on performance in the general knowledge task. This included the questions “What do you think the purpose of this pair of experiments was?” and “Do you think the previous exercise where you had to list words related to a typical professor had any effect on all of the ratings you have given?”

Procedure. Participants were seated in a cubicle containing a computer and desk. They were told that they would take part in two separate experiments. In addition, the data that would be collected from these two experiments were said to be for different research groups which would be used for forthcoming experiments of those groups in the future.

*Results*

The majority of participants were not aware of the purpose of the study: only 5/44 participants (11%) reported that the priming manipulation affected the way they performed in the general knowledge task. Furthermore, these reports included some participants (3) who believed that the hooligan attribute listing procedure helped them concentrate better in the knowledge test.

**Experiment 4**

*Methods*

Participants. One hundred students from the University of New South Wales participated in the experiment for course credit. There were 34 males and the mean age was 19.4 (*SD* = 2.8).

Materials. The experiment was computer-based and similar (unless otherwise noted) to Experiment 3. Participants were tested in individual cubicles. A new sample of 40 general knowledge questions was drawn from various sources and the order of questions was randomized for each participant.

Procedure. The task instructions were as follows:

“Thankyou for agreeing to take part in this study. All of the instructions will be presented on the computer. Imagine a typical professor [soccer hooligan]. Please list the behaviours, lifestyle, and appearance attributes of this professor [soccer hooligan] in the space provided below. You will be given 5 minutes to do this. The computer will let you know when to start and stop. This information will be used by researchers in the Social Psychology Lab.”

After completing their response on a page of paper, the instructions continued: “This task is complete. Please press Continue to save your answer and go on to the next stage. The Personality Research Group is developing a general knowledge scale. This scale consists of 5 subscales, each containing 40 questions. The subscales range from (1) very easy to (5) very difficult and we would like to test the differences in difficulty between these. You are being given the most difficult test among the 5 sets. You will be given 40 general knowledge questions to do. Each question will appear on the screen. Choose an answer by clicking on the button next to it. Then, click continue to confirm your answer and proceed to the next question. You must answer every question and if you do not know the answer for the question try your best to guess. There is no time constraint.”

At the end of the experiment a questionnaire was administered to test participants’ awareness of any influence of the priming procedure on performance in the general knowledge task. This included the following items: “1. You described the characteristics of a professor [soccer hooligan]. Can you recall which Lab is collecting these data? 2. You answered some general knowledge questions. Can you recall which Research Group is collecting these data? 3. Do you think your listing of the characteristics of a professor [soccer hooligan] might have affected your performance in answering the general knowledge questions. If you do, then in what way?”

*Results*

The majority of participants were not aware of the purpose of the study: only 16/100 participants (16%) reported that the priming manipulation affected the way they performed in the general knowledge task. Most reports were nonspecific; only 8 of the reports approximated awareness of the experimental hypothesis. Performance in the two groups on answering the general knowledge questions was not qualitatively affected by removal of these participants.

**Experiment 5**

The study was advertised as trying to collect data for various research projects. Further, in order to enhance the illusion that data were in fact being collected for different studies, a confederate was used to administer the general-knowledge test while the researcher administered the priming manipulation. Also, different media were used for the different tasks - after an unrelated filler task conducted at a computer, the priming manipulation consisted of participants writing down similarities/differences to professors/Einstein respectively in a questionnaire printed on paper and the general-knowledge test was administered at a computer.

*Methods*

Participants. 49 participants were recruited from the UCL Psychology subject pool and received payment of £3. Only people who were not currently or at some point Psychology students were recruited for the study. They were randomly assigned to one of two priming conditions (professor-similarities/Einstein-differences) in a between-groups design. The sample consisted of 28 female and 21 male participants with a mean age of 25.5 years (*SD* = 5.7).

Materials. The filler task was a simple modification of a Stroop test used for collecting reaction times of the participants upon the appearance of a target stimulus on the screen. This was programmed using PsychToolbox in MATLAB and was administered at a computer. The priming manipulation questionnaires were designed to be similar to the ones used in LeBoeuf and Estes’ study [[22](#_ENREF_22)]. The participants in the professor-similarity condition were instructed, “List three ways in which you are similar to a professor”. For participants in the Einstein-differences condition, the words “similar to” were replaced with “different from” and “Albert Einstein” replaced the words “a professor”.

The general-knowledge test consisted of 20 multiple-choice questions, each question having four answer choices, and was administered via computer. The questions were modelled after LeBoeuf and Estes’ study and had the same difficulty level. The order of the questions and the answer choices were randomised.

Procedure. Participants were recruited for what they believed was a data-collection programme run by the department for various on-going research projects. The instructions for the first task, which unknown to them was a filler task, were presented. The filler task was a simple modification of a Stroop test. When participants finished this task, they proceeded to the priming questionnaire. Participants were asked to list their details (name, age, sex) for each study, so as to heighten the illusion that they were participating in different studies. After completion of this questionnaire, a confederate who was supposedly running the third study presented the instructions for the general knowledge test.

When participants finished the task, the researcher handed them the funnel-debriefing questionnaire. The first page asked the participants, “One of the tasks you just completed was a multiple-choice trivia test. What do you think the purpose of this task was?” The participants then moved on to the next page which presented the question, “Do you think the tasks presented to you were related in any way?” The next question asked them to specify how the tasks were related if they had answered “Yes” to the previous question. On the next page, they were asked if they thought that the similarities/difference listing task might have affected their performance in the general knowledge test, and if so, how.

*Results*

Based on the debriefing, one participant indicated that she had insight about the study’s purpose.

**Experiment 6**

*Methods*

Participants. 32 participants were recruited from the UCL Psychology subject pool and received payment of £3. They were randomly assigned to one of two priming conditions (professor-similarities/Einstein-differences). Only non-Psychology students were recruited for the study. The sample consisted of 15 female and 17 male participants and the mean age was 27.3 years (*SD* = 12.1).

Materials. The materials used were the same as those in Experiment 5.

Procedure. Participants were informed that they would participate in two studies to facilitate data-collection for ongoing studies in the department, a reaction time study (the modified Stroop test) and the priming study. This time, however, the priming manipulation and the general knowledge test were described as being part of the same study and hence no confederate was required. The first task was the modified Stroop test. On completion of this, they were told that the next study comprised two parts. They were instructed that they would be asked to fill out a questionnaire which would make them list similarities between themselves and professors or differences between themselves and Einstein. They were then told that the researchers were interested in seeing if making them compare themselves to professors would impair performance on a subsequent general knowledge test. In the Einstein-differences condition, they were told that the researchers were interested in seeing if making them think about Einstein and hence intelligence would improve their performance.

After the participants completed the priming manipulation questionnaire, which was the same as that used in the previous study, the general knowledge test was presented. The same questions were used as in the previous study. Finally, they completed a debriefing questionnaire which contained the same questions as before, but this time participants were expected to believe that the studies were indeed related, as they had been told so beforehand. Participants were also questioned to check if they remembered the direction in which they had been told their performance in the general knowledge test would be affected by the priming manipulation. This was done in order to check if they had understood what the studies were about and how their performance would be influenced.

**Experiment 7**

*Methods*

Participants and design. Forty-eight UCL students participated in the experiment (no psychology students were included). Twenty-three were male and the mean age was 23.1 (*SD* = 3.9). Participants were randomly allocated to one of the experimental conditions of a 2 (social identity of comparison target: in-group vs. out-group) x 2 (intelligence of comparison target: professor vs. soccer hooligan) factorial design.

Procedure. For the priming manipulation, the experiment was introduced as a study about impression formation. Participants were given a booklet which contained this exercise. It was explained that the goal of the study was to investigate the social perception and judgment of students in large cities. To activate an intergroup focus, participants (themselves students from London) were informed that students from London are likely to give more accurate descriptions of each other than students from Birmingham because London students are more likely to have met each other socially. In addition, it was stated that their results would be compared with the results of the Birmingham students, where the same experiment was being run by the psychology department of Birmingham University.

In the impression formation exercise, participants were asked to form an impression of a person who was described as either a professor or soccer hooligan for the manipulation of the intelligence of the comparison target. For example, in the professor condition, M was described as a person who only received A’s for his undergraduate degree, went on to do more research and became a professor. For the soccer hooligan condition, M was described as a lazy student who failed his year twice during the undergraduate degree, was violent and became a member of a big soccer hooligan gang. There were 20 such descriptions about the target person. For the manipulation of the social identity of the target, the person was described as either a former student from UCL or from Birmingham University.

After the priming procedure, participants were asked to rate the person described on several personality traits, some of which were related to the way we manipulated intelligence (i.e., “M is ambitious with his work.”, “M is intelligent”, “M likes to find answers to problems”, etc.). Items that were not related to intelligence were also included (e.g., “M is an outgoing person”, “M is a confident person”, “M is optimistic”, etc.). These personality trait items were rated on 5-point scales (1 = strongly disagree, 5 = strongly agree) and were included to confirm the selectivity of the manipulation of intelligence. Next, participants were given a purportedly unrelated knowledge test as a booklet of 15 multiple choice general knowledge questions.

After completing the test, participants were presented with a post-experiment funnel questionnaire to assess some of the motivational components that may have guided their behaviour as well as their awareness of the link between the priming manipulation and the general knowledge test.

*Results*

A manipulation check of intelligence of the comparison target was undertaken. A 2 (intelligence of target) x 2 (identity of target) ANOVA on the combined items that were related to intelligence in the impression formation exercise showed that person M was rated as more intelligent in the high intelligent target conditions (*M* = 4.41, *SD* = 0.06) than in the low intelligent target conditions (*M* = 2.24, *SD* = 0.06), *F*(1, 44) = 608.51, *p* < 0.001. This suggests successful manipulation of inferred intelligence.

One participant volunteered that the priming manipulation and the general knowledge test were related. Asked specifically whether there might have been an influence, 9 participants responded affirmatively but 6 of these guessed the wrong direction of effect.

**Experiment 8**

*Methods*

Participants and design. Eighty subjects from the UCL community participated in the experiment. Forty-seven were female and the mean age was 25.7 (*SD* = 7.8). Participants were randomly allocated to one of the four conditions: incentive, no incentive, professor prime, and hooligan prime, and tested individually. Those in the prime conditions were paid £3, while those in the incentive condition could earn between 50 pence and £8.50 depending on the number of questions answered correctly. Participants in the no incentive condition were paid 50 pence.

Procedure. The general knowledge test was similar to that used in Experiment 4 with 40 general knowledge 4-alternative multiple choice questions in total. Participants in the professor and soccer hooligan prime conditions completed self-monitoring and self-consciousness questionnaires after the knowledge test, and finally a funnel debriefing.

**Experiment 9**

*Methods*

Participants. 66 student community volunteers participated in the study (mean age 28.1, *SD* = 7.8, 40 males, 26 females). Half were from Greece and half from the United Kingdom, approximately evenly distributed across conditions.

Design. Participants were randomly assigned to one of the six conditions of a 2 (self-construal: independence vs. interdependence) x 3 (primed category: Professors vs. Hooligans vs. control) between-subjects design.

Materials. The experimental procedure consisted of three different tasks. The first task presented to participants as a personality questionnaire was the self-construal manipulation. Seven items [[53](#_ENREF_53)] were presented and participants evaluated how well each item described them on a 7-point scale (1= totally disagree to 7= totally agree).

In the second task, participants were presented with a positive (professor) or a negative (hooligan) stereotype priming task (this stage was omitted for no-prime control subjects). In the professor priming task, participants were presented with 10 pictures representative of this stereotype and answered questions related to them (e.g., “What is his profession?”, “What might this person be thinking about?”). The same procedure was used in the hooligan stereotype but with different pictures and questions (e.g., “Why are the police trying to arrest these people”, “Why are these people fighting?”).

The third task was a general knowledge test comprising 20 questions each with 3 response options. We split the questions into 4 categories (difficult, moderately difficult, moderately easy, and easy) according to the percentage of correct answers for each question.

Procedure. At the beginning of the experiment participants were told that there was no link between the three tasks. Participants in the experimental conditions completed three tasks. They first answered the self-construal questionnaire (either interdependent or independent) on paper, then sat in front of a computer screen and observed pictures (either professors or hooligans) and answered the questions on an answer sheet provided by the experimenter. Finally they were instructed to complete the general knowledge task on paper. In the control condition, participants were first given the self-construal manipulation questionnaire (either interdependent or independent) and then they completed the general knowledge task on paper.

*Results*

We estimated the mean number of correct responses for each of the 20 questions and ranked them into 4 groups of 5 questions according to their difficulty. The questions were categorized as difficult (47% correct), moderately difficult (61% correct), moderately easy (79% correct), or easy (92% correct).

In addition to the analysis on the difficult questions, we examined all questions combined. The means (*SD*s) were as follows. Independent self-construal: professor prime *M* = 75.0 (17.7), no prime *M* = 71.0 (10.7), hooligan prime *M* = 68.6 (13.1); Interdependent self-construal: professor prime *M* = 64.3 (14.0), no prime *M* = 68.8 (17.3), hooligan prime *M* = 72.8 (15.8). A 2 (self-construal: independence vs. interdependence) x 3 (professor stereotype, hooligan stereotype, no prime condition) ANOVA revealed nonsignificant main effects of self-construal, *F*(1, 60) = 0.62, *p* = .44, and prime, *F*(2, 60) = 0.03, *p* = .97. The interaction was also non-significant, *F*(2, 60) = 1.32, *p =* .28. As with the subset of difficult questions, the professor-hooligan differences are in the opposite direction to those observed by Bry et al. [[20](#_ENREF_20)].
